# Short- *versus* Long-Sarafotoxins: Two Structurally Related Snake Toxins with Very Different *in vivo* Haemodynamic Effects

**DOI:** 10.1371/journal.pone.0132864

**Published:** 2015-07-15

**Authors:** Yazine Mahjoub, Stéphanie Malaquin, Gilles Mourier, Emmanuel Lorne, Osama Abou Arab, Ziad A Massy, Hervé Dupont, Frédéric Ducancel

**Affiliations:** 1 Pôle d’anesthésie, réanimation et médecine d’urgence, CHU Amiens, Amiens, France; 2 Unité INSERM U1088, Amiens, France; 3 CEA, iMETI, Service d’Immuno Virologie (SIV), CEA Fontenay-aux-Roses, F-92265 Fontenay-aux-Roses, France; 4 CEA, iBiTec-S, Service d’Ingénierie Moléculaire des Protéines (SIMOPRO), CEA Saclay, F-91191 Gif sur Yvette, France; Universidade Federal do Rio de Janeiro, BRAZIL

## Abstract

Sarafotoxin-m (24 amino acids) from the venom of *Atractaspis microlepidota microlepidota* was the first long-sarafotoxin to be identified, while sarafotoxin-b (21 aa) is a short-sarafotoxin from *Atractaspis engaddensis*. Despite the presence of three additional C-terminus residues in sarafotoxin-m, these two peptides display a high sequence homology and share similar three-dimensional structures. However, unlike sarafotoxin-b, sarafotoxin-m shows a very low *in vitro* affinity for endothelin receptors, but still has a very high *in vivo* toxicity in mammals, similar to that of sarafotoxin-b. We have previously demonstrated, *in vitro*, the crucial role of the C-terminus extension in terms of pharmacological profiles and receptor affinities of long- *versus* short-sarafotoxins. One possible hypothesis to explain the high *in vivo* toxicity of sarafotoxin-m could be that its C-terminus extension is processed *in vivo*, resulting in short-like sarafotoxin. To address this possibility, we investigated, in the present study, the *in vivo* cardiovascular effects of sarafotoxin-b, sarafotoxin-m and sarafotoxin-m−Cter (sarafotoxin-m without the C -terminus extension). Male Wistar rats were anaesthetised and mechanically ventilated. Invasive haemodynamic measurements and echocardiographic measurements of left and right ventricular function were performed. The rats were divided into four groups that respectively received intravenous injections of: saline, sarafotoxin-b (one LD_50_), sarafotoxin-m (one LD_50_) or sarafotoxin-m−Cter (one LD_50_). All measurements were performed at baseline, at 1 minute (+1) and at 6 minutes (+6) after injection. Results: Sarafotoxin-b and sarafotoxin-m-Cter decreased cardiac output and impaired left ventricle systolic and diastolic function, whilst sarafotoxin-m decreased cardiac output, increased airway pressures and led to acute right ventricular dilatation associated with a decreased tricuspid annulus peak systolic velocity. Sarafotoxin-b and sarafotoxin-m−Cter appear to exert toxic effects via impairment of left ventricular function, whilst sarafotoxin-m increases airway pressures and impairs right ventricular function. These results do not support the hypothesis of an in vivo processing of long sarafotoxins.

## Introduction

Sarafotoxins (SRTXs) extracted from the venom of snakes belonging to the genus *Atractaspis* and endothelins synthesised by mammalian endothelial cells belong to the same family of endothelin-like peptides [1]. Human endothelin-1, as well as sarafotoxin-b (SRTX-b) extracted from the venom of *Atractaspis engaddensis*, are considered to be the most potent vasoconstrictors described to date [2,3]. These two peptides are 21 amino acids long and stabilised by two disulfide bridges between common cysteines +1/+15 and +3/+11. They interact with endothelin receptors (ET-A and ET-B) situated on the membrane of various cells [4,5]. SRTX-b shows a high sequence homology with endothelin-1 as well as a similar three-dimensional structure [6]. Long SRTXs, including SRTX-m, were discovered in the venom of *Atractaspis microlepidota microlepidota* [7]. SRTX-m has a longer C-terminus extension with three additional residues “D-E-P”. Solution structures of SRTX-b and-m have revealed well-defined and conserved C1-C15 domains [6]. All these endothelin-like peptides share the same 3D structure with an extended structure of the four N-terminal amino acids, a bend between positions +5 and +8, and an alpha-helical conformation of the segment Lys9-Cys-15 [8]. In contrast, the C-terminus extension of SRTX-b (residues +16/+21) is conformationally flexible and disordered, while in SRTX-m, the conformation of the long C-terminus tail is more restricted. The presence of the C-terminus extension determines the affinity and selectivity towards endothelin-receptors, as SRTX-m shows a very low affinity for ET-A and ET-B human receptors. Surprisingly, and despite these differences, the *in vivo* toxicity of SRTX-m in the mouse is very similar to that of SRTX-b [7]. It has been hypothesised that endogenous maturation by specific endoproteases of the prey, may remove the longer C-terminus extension of SRTX-m, resulting in a toxic effect similar to SRTX-b [6]. To assess this hypothesis, in this study we investigated the *in vivo* cardiovascular effects of SRTX-b, SRTX-m and SRTX-m−Cter, a C-terminus truncated form of SRTX-m. In order to precisely investigate cardiovascular effects, we combined invasive haemodynamic measurements and Doppler echocardiography. We investigated global haemodynamic parameters and also specific left and right ventricular function parameters.

## Materials and Methods

### Peptide synthesis

Sarafotoxin-b was purchased from Sigma (St Quentin Fallavier, France). Sarafotoxin-m and Sarafotoxin-m−Cter were synthesized using an automated chain assembly with a standard Applied Biosystems 433 peptide synthesizer, as previously described [6,9]. Sarafotoxins were synthesized on a Fmoc-Trp(Boc)-wang resin using standard solid phase synthesis techniques. Cysteines were introduced as Fmoc-Cys(Trt)-OH. Dicyclohexylcarbodiimide and 6-Chloro-1-hydroxybenzotriazole were used as coupling reagents. Peptides were separated from the resin after TFA deprotection. For each peptide, the crude material was purified by HPLC using a C18 column with an 18–30% CH_3_CN gradient in 0.1% TFA in water. Peptide reduced forms were subjected to an oxidative reaction in 0.1 M Tris/1mM EDTA buffer containing 0.5–2 M guanidine hydrochloride in the presence of reduced (GSH) and oxidized (GSSG) glutathione in a peptide:GSSG:GSH molar ratio of 1:10:100 at a peptide concentration of 0.05 mg/ml [6,9]. After 36 hours at 4°C, oxidized forms of the toxins were purified by HPLC and characterised by amino acid analysis using an automatic analyser (Applied Biosystem 130 A) and mass spectrometry on a Nermag spectrometer coupled to an analytical electrospray source.

### Animals

Seven-week-old male Wistar rats (360–410g) were maintained in a temperature- and humidity-controlled room with 12h light-dark cycle. They were given standard chow and had been made to fast for 12h before the experiment with *ad libitum* access to water.

### Ethics statement

Animal experiments were performed in accordance with the recommendations of the EU and the French National Committee for the care and use of laboratory animals. The institutional Animal Care Ethics committee of the Amiens University School of Medicine approved the experimental protocol (CREMEAP no 161112–16).

### Haemodynamic measurements

Animals were placed in the anaesthesia chamber containing 3% isoflurane for induction. Animals were then tracheotomised and mechanically ventilated in FiO_2_ = 100% with a tidal volume of 6.2 M^1.01^ mL and a respiratory rate of 53.5 x M^−0.26^ min^-1^ (M = animal weight in kg) [10]. Respiratory rate was adjusted to PaCO_2_ of 30–40 mmHg. Anaesthesia was maintained by inhaled isoflurane (1–1.5%) and analgesia was ensured by intraperitoneal (IP) injection of 1mg/kg of morphine. Adequate anaesthesia/analgesia was regularly checked (no response to tail pinch) and 20% of the initial dose of morphine was injected every 40 minutes. In order to avoid spontaneous breathing that may modify heart–lung interactions [10], pancuronium bromide (2mg/kg) was injected IP to induce muscle relaxation after checking for adequate anaesthesia and analgesia. Body temperature was maintained at 37–37.5°C with a heating pad.

A fluid-filled catheter was placed in the abdominal aorta via the femoral artery. A fluid-filled catheter was introduced via the left jugular vein into the right atrium to measure central venous pressure (CVP). A catheter-tipped transducer (Millar Instruments Inc, Houston, USA) was introduced into the left ventricle (LV) via the right carotid artery. Arterial, venous and LV pressures, as well as airway peak pressure were recorded simultaneously with a data acquisition system (IOX, EMKA Technologies). Maximal +dP/dt (+dP/dt_max_), and Tau, the relaxation time constant, were calculated simultaneously from the LV pressure curve to assess left ventricular systolic and diastolic function [11–12].

### Echocardiography

Transthoracic echocardiography measurements were performed by the same investigator in the left lateral decubitus position using an echocardiography device (Sonos 4500, Agilent Technologies) with a 7.5 MHz probe. Transthoracic echocardiography was performed simultaneously with the invasive haemodynamic measurements (i.e. under the same anaesthesia protocol). The left ventricular outflow tract diameter (D) was measured from the parasternal long-axis view. All measurements were averaged through four cardiac cycles. Left ventricular end-diastolic (LVEDA) and end-systolic (LVESA) areas were measured on the parasternal short-axis view, and the fractional area (LVFA) was calculated as follows: LVFA = 100 x (LVEDA-LVESA)/LVEDA

The right ventricular end-diastolic area (RVEDA) and the left ventricular end-diastolic area (LVEDA) were measured on the apical four-chamber view and the RVEDA/LVEDA ratio was calculated. The time between closure and opening of the mitral valve (a) was recorded. The velocity-time integral of aortic flow (VTIAo) was measured on the apical 5-chamber view using pulsed Doppler with the smallest sample volume placed at the level of the aortic annulus. Stroke volume (SV) and cardiac output (CO) were calculated as SV = VTIAo x 3.14 x D^2^/4 and CO = SV x HR where HR = heart rate. Systemic vascular resistance (SVR) was calculated as SVR = (MAP-CVP)/CO, where MAP = mean arterial pressure. The duration of aortic flow was measured (b) and the left ventricular myocardial performance index (Tei index) was calculated as previously described: Tei index = (a-b)/b. [13]. The tricuspid annulus peak systolic velocity (St) was recorded by tissue Doppler imaging to assess right ventricular systolic function.

### Experimental protocol

A 45-min stabilization period was observed after completion of the preparation. Using a random numbers table, animals were randomly allocated to four groups to receive saline (saline group), SRTX-b (SRTX-b group), SRTX-m (SRTX-m group) or SRTX-m−Cter (SRTX-m−Cter group). In order to observe marked toxic effects similar to those that lead to the death of poisoned prey, each toxin was administered at a dose of one LD_50_ that corresponded to 15 μg.kg^-1^ for SRTX-b, 32 μg.kg^-1^ for SRTX-m and 15 μg.kg^-1^ for SRTX-m−Cter [3,7]. All doses were diluted in one mL of saline and injected through the left jugular vein over one minute. Based on previous experiments showing a very acute lethal effect of sarafotoxins (within eight minutes), all measurements were performed at baseline and repeated 1 and 6 minutes after injection of the toxin [7].

At the end of the experiment, the animals were euthanized with an intravenous dose of sodium pentobarbital.

### Statistical analysis

Data are expressed as the median with interquartile range (IQR). An ANOVA test for repeated measures analysis of variance was performed for comparisons over time between and within groups. One-way ANOVA analysis of variance, followed by a Student–Newman–Keuls *post hoc* test, was performed for pairwise comparisons. A *p*-value<0.05 was considered statistically significant. Intra-observer reproducibility of echocardiographic parameters was assessed in ten rats: echocardiography was performed twice on each rat with a one-hour interval between measurements. Reproducibility was defined as the mean difference between two measurements, expressed as a percentage (absolute difference divided by the average of the two observations).

## Results

Fifteen rats were allocated to the saline group, 16 to the SRTX-b group, 13 to the SRTX-m group and 12 to the SRTX m-Cter group.

### Reproducibility of echocardiography measurements

Intra-observer reproducibility of the Tei index, RVEDA/LVEDA, St, VTIAO and the LVFA was 8±1%, 10± 2%, 5±0.5%, 9±1% and 10±1% (n = 10 each) respectively.

### Global haemodynamic parameters and airway pressure

**Fig 1:**
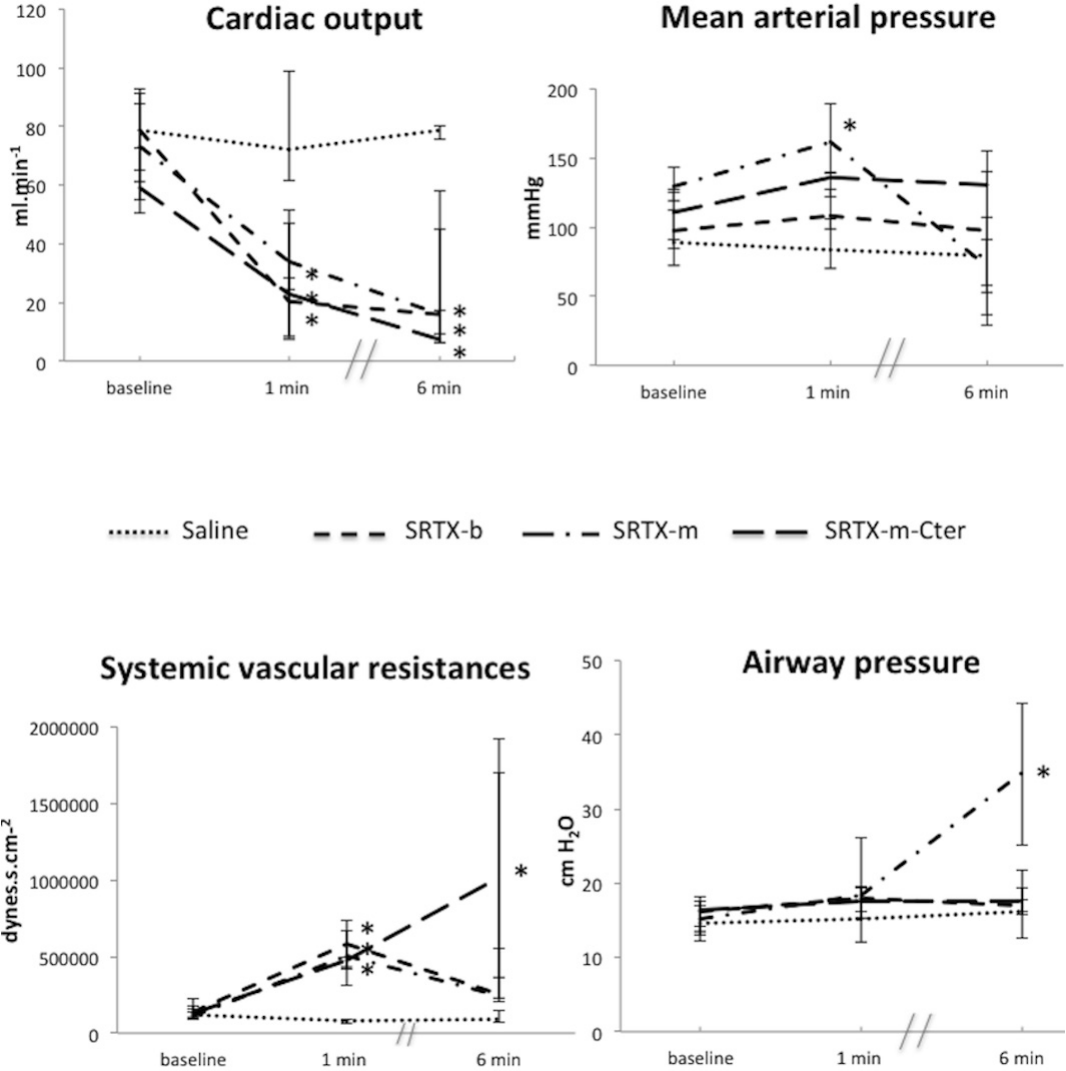
Effects of the toxins on global haemodynamic indices and airway pressure. Toxins or saline were perfused at baseline. Haemodynamic measurements were recorded at baseline and at 1 min and 6 min post-toxin. The dots represent the median and interquartile range. *n* = 15 for Saline group; *n* = 16 for SRTX-b; *n* = 13 for SRTX-m; *n* = 12 for SRTX–m-Cter. *p<0.05 versus baseline

The Heart rate was slightly decreased for all toxins (Table 1). However, the relative change in heart rate for each group was the same (between 8 and 10%), even for the saline group, suggesting that toxins do not cause a substantial alteration in heart rate, but that changes are probably due to the preparation by itself. Mean arterial pressure remained unchanged over time for SRTX-b and-m-Cter whereas the SRTX-m group showed a transient increase in mean arterial pressure after 1 minute (Fig 1). Cardiac output showed a significant and sustained decrease with all toxins. SVR increased for all toxins at 1 minute and continued to increase with SRTX-m-Cter at 6 minute. Airway pressure showed a marked increase at 6 min in the SRTX-m group, but remained unchanged for the other toxins.

**Table 1:** Heart rate variations for each toxin group.

	Baseline	t = +1 min	t = +6 min
HR (bpm)			
Saline	433 [397–447]	429 [390–444]	391 [372–421]
SRTX-b	397 [381–412]	371 [355–405]* §	357 [346–370]* §
SRTX-m	400 [377–411]	377 [359–398]	367 [319–377]*
SRTX-m-Cter	395 [376–444]	371 [344–388]* §	363 [348–424]* §

Data are expressed as the median and interquartile range [IQR].

*p<0.05 vs Baseline

§p<0.05 vs Saline at the same time

### LV function

**Fig 2:**
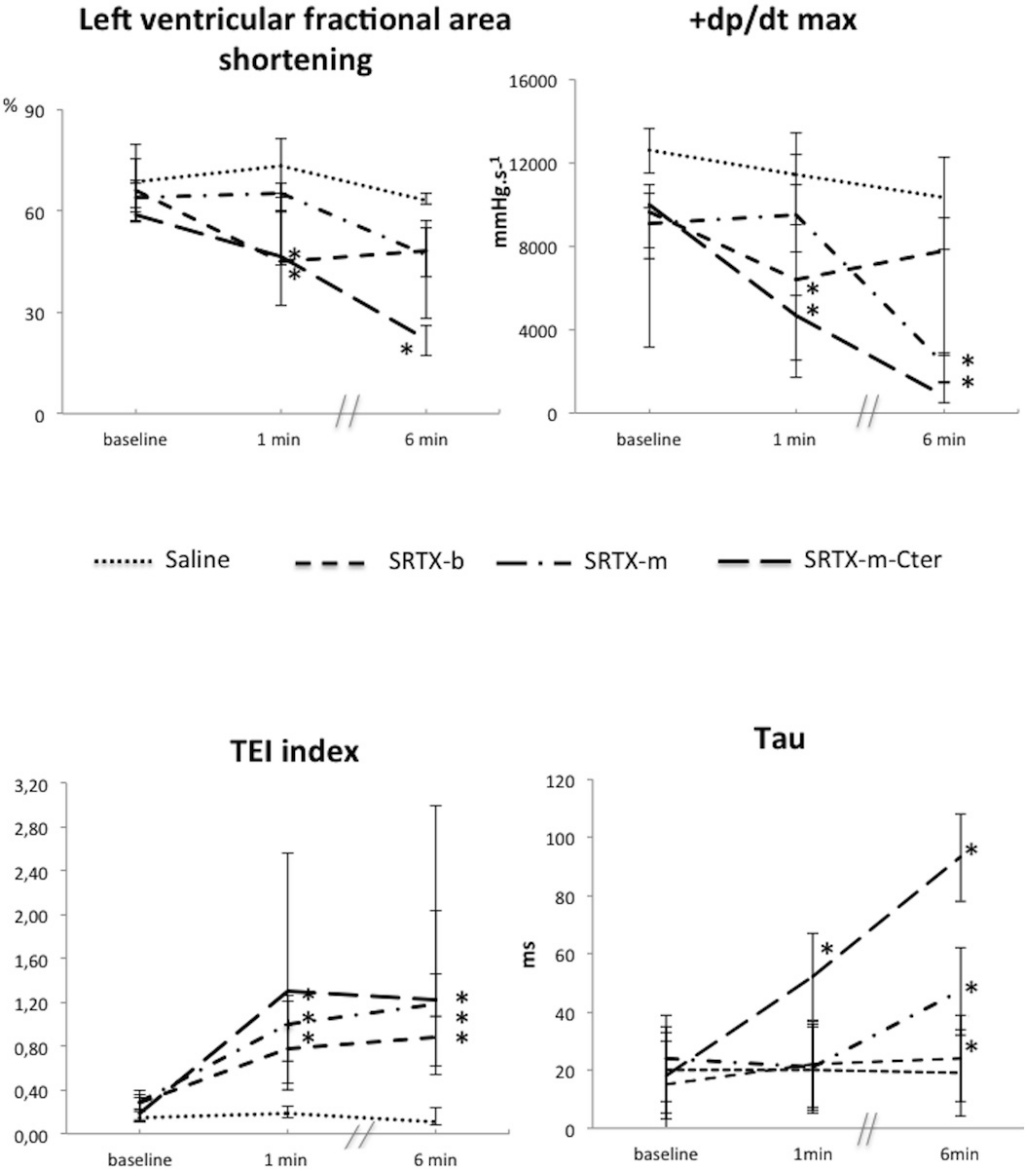
Effects of the toxins on left ventricular function indices. Toxins or saline were perfused at baseline. Haemodynamic measurements were recorded at baseline, and at 1 min and 6 min post-toxin. The left ventricular area shortening and Tei index were recorded using Doppler echocardiography. +dp/dt max and Tau were recorded using a catheter placed in the left ventricle. The decrease in left ventricular area shortening and +dp/dt max indicated impairment of left ventricular systolic function. The increased Tei index reflected impairment of global left ventricular function, whereas the increase in Tau indicated impairment of left ventricular diastolic function. The dots represent the median and interquartile range. *n* = 15 for Saline; *n* = 16 for SRTX-b; *n* = 13 for SRTX-m; *n* = 12 for SRTX–m-Cter. *p<0.05 versus baseline

LV systolic function was evaluated invasively by +dp/dt max, and noninvasively by LVFA. The two measurements were concordant and showed early impairment of LV systolic function for SRTX-b and SRTX-m-Cter (at 1 minute) that persisted with the latter at 6 min; delayed impairment (at 6 min) was seen for SRTX-m (Fig 2). LV diastolic function assessed by the Tau relaxation time constant showed early impairment (increased Tau duration) with SRTX-m-Cter and delayed impairment for all toxins (Fig 2). However, the three toxins did not produce the same degree of impairment: indeed the decrease in Tau is more marked for SRTX-m-Cter than for the other toxins. Notably, the difference between the responses to SRTX-b and SRTX-m-Cter was approximately 5-fold. In addition, the increase in Tau with SRTX-b at 6 min was very slight. Global LV function (diastolic and systolic), as assessed by the Tei index, showed an early decrease with all toxins (Fig 2).

### RV function

**Fig 3:**
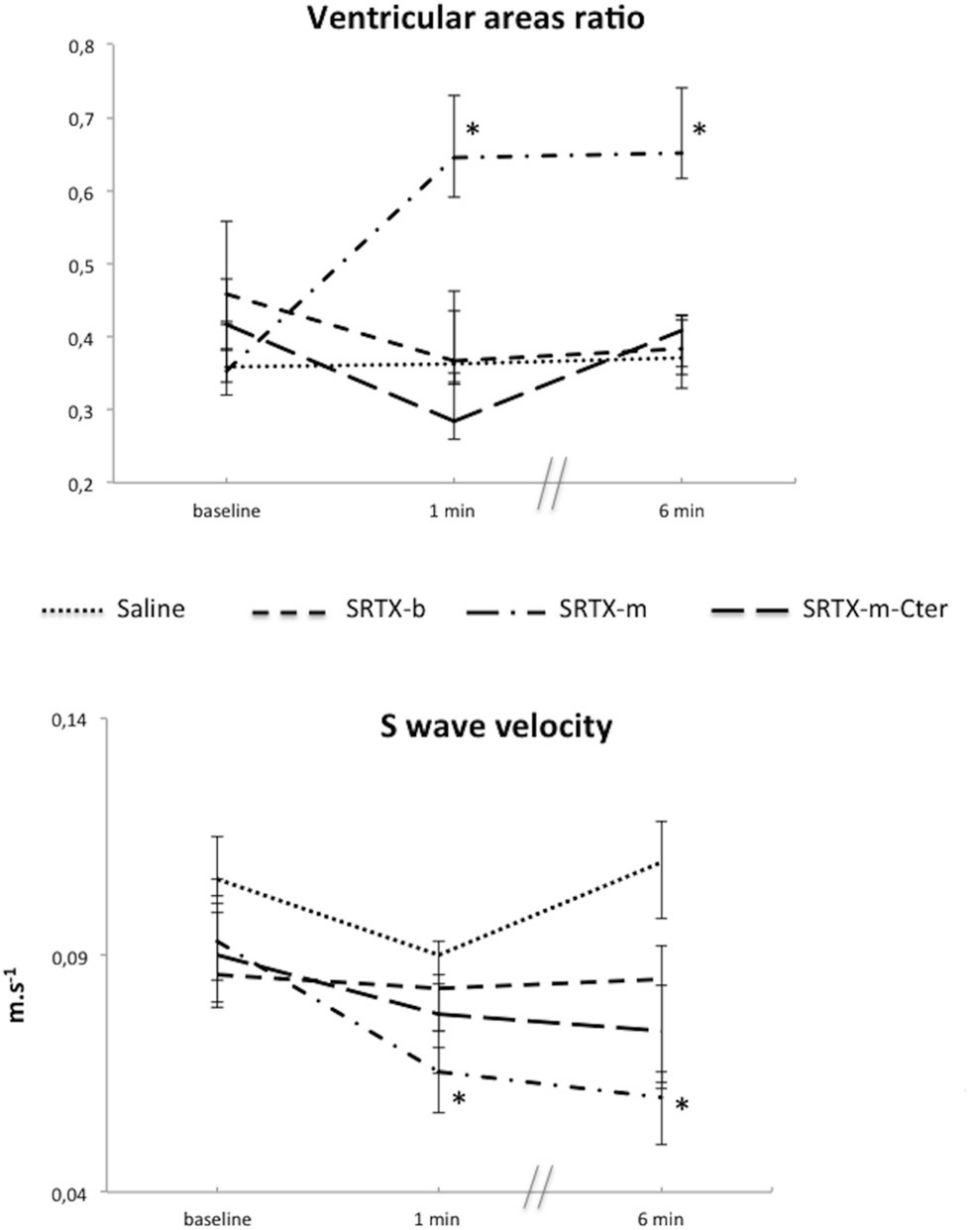
Effects of the toxins on right ventricular function indices evaluated by Doppler echocardiography. Toxins or saline were perfused at baseline. Haemodynamic measurements were recorded at baseline and at 1 min and 6 min post-toxin. The ratio of the right ventricle diastolic area to the left ventricle diastolic area increases in case of acute right ventricular dilatation. This dilatation is a sign of right ventricular dysfunction. The peak systolic velocity of the tricuspid annulus (S wave) is a parameter of right ventricular systolic function. A decrease in this velocity is consistent with a right ventricular systolic dysfunction. The dots represent the median and interquartile range. *n* = 15 for Saline; *n* = 16 for SRTX-b; *n* = 13 for SRTX-m; *n* = 12 for SRTX–m-Cter. *p<0.05 vs baseline

RV function was assessed by two parameters: RVEDA/LVEDA ratio [14] and St peak systolic velocity [15]. RVEDA/LVEDA increased significantly at 1 min and 6 min only in the SRTX-m group, corresponding to acute RV dilatation (Fig 3). Moreover, St peak systolic velocity decreased significantly over time only in the SRTX-m group, reflecting impairment of RV systolic function (Fig 3).

The changes observed with each toxin are summarized in Table 2.

**Table 2:** Summary of changes observed with each toxin.

			Toxin	
Parameter		SRTX-b	SRTX-m	SRTX-m-Cter
CO (ml.min-^1^)	1min	↓	↓	↓
	6 min	↓	↓	↓
MAP (mmHg)	1 min	↔	↑	↔
	6 min	↔	↔	↔
SVR (dynes.s.cm^-2^)	1 min	↑	↑	↑
	6 min	↔	↔	↑
Airway pressure (cmH_2_O)	1 min	↔	↑	↔
	6 min	↔	↑	↔
LVFAS (%)	1 min	↓	↔	↓
	6 min	↔	↔	↓
+dp/dt max (mmHg.s^-1^)	1 min	↓	↔	↓
	6 min	↔	↓	↓
Tei index	1 min	↑	↑	↑
	6 min	↑	↑	↑
Tau (ms)	1 min	↔	↔	↑
	6 min	↑	↑	↑
RVEDA/LVEDA	1 min	↔	↑	↔
	6 min	↔	↑	↔
St Velocity (m.s^-1^)	1 min	↔	↓	↔
	6 min	↔	↓	↔

↑ = increase ↓ = decrease ↔ = no change

CO = cardiac output; MAP = mean arterial pressure; SVR = systemic vascular resistance; LVFAS = left ventricular fractional area shortening; RVEDA = right ventricular end diastolic area; LVEDA = left ventricular end diastolic area; St = tricuspid annulus systolic wave

## Discussion

In this study, we observed that the acute haemodynamic effects of the long-sarafotoxin, SRTX-m, differed from those of the short-sarafotoxin SRTX-b. SRTX-m increased airway pressures and impaired RV function, while SRTX-b impaired LV function with no effect on airway pressures. Of note, we also found that the truncated isoform SRTX-m-Cter had very similar effects to those of SRTX-b, highlighting the fact that the C-terminus extension of these peptides is responsible for substantial *in vivo* physiological effects.

*Atractaspis* snakes are venomous snake from the Middle East and Africa and envenomation from *A*. *engaddensis* (the burrowing asp) is responsible for skin necrosis and possibly fatal cardiac conduction disorders [16,17]. SRTX-b is a major active component of this venom. In mammals, endothelin-1 is synthesised by endothelial cells and plays a major physiological role in cardiovascular homeostasis [18]. These peptides interact with G protein-coupled receptors expressed by on various mammalian cell membranes [19]. Two main types of receptors have been described to date: ET-A and ET-B. Endothelin binding to ET-A and ET-B receptors of smooth muscle cells is responsible for vasoconstriction, while endothelin binding to ET-B receptors of endothelial cells is responsible for vasodilatation via NO and icosanoid release [20,21].

SRTX-m from the venom of *A*. *m*. *microlepidota* is considered to be a “long-sarafotoxin” [7]. It presents a high homology with SRTX-b, as 81% of the first 21 common amino acids are identical. In addition, three of the four substitutions are conservative: Met/Ile, Asn/Thr and Met/Leu at positions +6, +7 and +12, respectively. The fourth amino acid substitution is located at position +4 with an Asn (SRTX-m) or Lys (SRTX-b). The main structural difference is a longer C-terminal domain in SRTX-m with “D-E-P” as additional amino acids. Further experiments have shown that SRTX-m is highly toxic, although slightly less toxic than SRTX-b [7]. Nevertheless, it has been demonstrated, on rabbit aortic rings, that the SRTX-m concentration necessary to induce half of maximum contraction is more than tenfold greater than that of SRTX-b (65 nM *vs* 5.6 nM) and that unlabelled SRTX-m is unable to displace labelled SRTX–b on rat atria cell membranes [7]. Moreover, Mourier et al. [6] showed, on CHO cells expressing ET-A and ET-B receptors, that the affinity of SRTX-m was four orders of magnitude lower than that of SRTX-b. Of note, truncation of the three C-terminus residues of SRTX-m, results in a marked increase in affinity of resulting SRTX-m-Cter for both ET-A and ET-B human receptors. In both cases an affinity increase of 3- to 4-orders of magnitude has been determined for SRTX-m-Cter to reach similar affinities than that of short-SRTX-b for these two major receptors. [6]. These authors hypothesised that *in vivo* processing by endogenous enzymes may remove the C-terminus extension of SRTX-m, thereby restoring its affinity for endothelin receptors. In the present study, we compared the acute hemodynamic effect (that is probably responsible for the toxic effect) of SRTX-b and SRTX-m to validate this hypothesis. In this case, we would expect a similar *in vivo* effect of SRTX-b and SRTX-m, but probably delayed by *in vivo* maturation. However, the present study did not confirm this hypothesis, as the effects of SRTX-m differed from those of SRTX-b. Moreover, the SRTX-m-Cter that could have resulted from endogenous processing of SRTX-m had a similar effect to that of SRTX-b, but different from SRTX-m. Finally, the effect of SRTX-m, like SRTX-b, was observed early (within 1 min), which is not consistent with endogenous maturation.

SRTX-b is responsible for an early decrease in cardiac output, LVFA, and dpt/dt max. These results may suggest an increase in LV afterload and are in accordance with previous studies. Han et al. [22] showed that *in vivo* infusion of SRTX-b increased total peripheral resistance and decreased cardiac output. We also found that SRTX-b exerted a negative lusitropic effect i.e impairment of LV relaxation as reflected by an increased Tau, the relaxation time constant. This finding is in accordance with previous animal and human studies [23,24]. Konrad et al. [24] studied the haemodynamic effect of systemic endothelin-1 (a peptide very similar to SRTX-b in terms of its structure and affinity for ET-A and ET-B receptors) infusion on anaesthetised pigs and found a similar effect consistent with increased LV afterload and impairment of relaxation. We also found that SRTX-m-Cter had very similar effects to those of SRTX-b, emphasising the fact that the C-terminus extension of these peptides has an important *in vivo* physiological effect. On the other hand, the acute *in vivo* haemodynamic effect of SRTX-m differed from that of SRTX-b and SRTX-m-Cter. SRTX-m is responsible for an early drop in CO, consistent with acute RV dysfunction. We used two parameters to assess RV function in this study: the RVEDA/LVEDA ratio and the tricuspid annulus peak systolic velocity (St). An increase in the RVEDA/LVEDA ratio reflects RV dilatation. Several clinical studies have shown that acute RV dysfunction due to increased RV afterload is responsible for acute RV dilatation [14,24,25]. Several studies have demonstrated that the tricuspid annulus peak systolic velocity (St) allows reliable evaluation of RV systolic function [15,24,26]. This effect was not observed with SRTX-b and SRTX-m-Cter. This RV dysfunction may be due to an acute increase in RV afterload. In support of this hypothesis, airway pressures were markedly increased with SRTX-m. This increase in airway pressure due to the collapse of small arteries and microvessels increases RV afterload [27,28,29].

Several hypotheses have been proposed to explain this bronchoconstrictor effect. Previous studies have shown that airway smooth muscle cells express ET-B and ET-A receptors and that binding to ET-A and ET-B receptors is responsible for a bronchoconstrictor effect [30, 31]. A low affinity for vascular smooth muscle cell ET-A receptors by lowering the vasoconstrictor effect of SRTX-m on pulmonary vessels, may facilitate access of the toxin to airway ET receptors, leading to bronchoconstriction [32, 33]. This phenomenon may be enhanced by the higher affinity of SRTX-m for ET-B receptors compared to ET-A receptors [6]. The vasodilator effect mediated by endothelial ET-B is therefore not fully reversed by the vasoconstrictor effect mediated by smooth muscle cell ET-A. Moreover, Jacques et al. [34] have shown that endocardial endothelial cells express ET-A receptors and that the density of these receptors is higher in the left ventricle than in the right ventricle. Another explanation is the presence of an atypical receptor different from ET-A and ET-B located on the bronchus or right ventricle and having a higher affinity for SRTX-m. This hypothesis has been previously proposed, but there is currently no evidence to support it [6]. SRTX-m did not impair LV systolic function, but adversely affected global LV function, as assessed by the Tei index. A prolonged Tei index despite preserved systolic function is consistent with diastolic dysfunction [13]. This diastolic dysfunction was confirmed by an increased Tau, the relaxation time constant. This impairment could be explained by RV dilatation. Since the pericardium is not able to expand despite RV dilatation, the LV is compressed and becomes unable to ensure adequate filling during diastole [35].

Our study presents a number of limitations. First, we cannot exclude the fact that the anaesthesia protocol may have had a significant effect on haemodynamic parameters. Nevertheless, we used a standard anaesthesia protocol to minimize this effect and we also included a sham group comprised of animals submitted to the same anaesthesia protocol [10]. Second, pressure-volume curves were not used to calculate load-independent LV performance indices such as end-systolic elastance and preload recruitable stroke work [36, 37], as the animals did not support acute preload change by simultaneous inferior vena cava compression and toxin infusion. However, Doppler echocardiography provided very accurate myocardial performance indices [38, 39]. Third, We did not investigate the effect of the toxins on coronary vessels. Previous studies have shown a marked vasoconstrictor effect of ET-1 or SRTX-b on coronary vessels that may lead to LV dysfunction [22,40]. However during the echocardiography procedure, no regional wall motion abnormalities were detected. Nevertheless, further investigation with coronary blood flow measurement and ischemia detection tools are needed to address this issue. Fourth, we used only a single dose of one LD50. It would have been of interest to evaluate the effect of different doses of each toxin. The high dose used here (one LD50) was chosen to specifically evaluate the mechanism of the lethal effect. Fifth, a longer forms of truncated SRTX-m with 22 or 23 amino acids have not been studied. These peptides may have different cardiovascular effect and deserves further study. Finally, the three dimensional structure of SRTX-m-Cter has not been charcaterised to date. However, as specified above, superposition of the NMR structures of SRTX isoforms reveals a conserved cysteine-stabilized-alpha-helical motif (residues 1–15) [7]. In the particular case of SRTX-m, the conformation of the long C-terminus tails (residues H16-P24) is found to be restricted owing to the nuclear Overhauser effect (NOE) connections between residues Y13 and F14 with the beginning of the C-terminus tail (side chains of Q17 and V19). These contacts strongly restrict the motions of the C-terminus extremity in SRTX-m. In SRTX-m-Cter, it is likely that the truncated C-terminus tail adopts the same conformation as that in SRTX-m, since truncation involves residues D22-E23-P24, which are not affected by any of the NOE connections observed in SRTX-m. Thus, the residues involved in the stabilising contacts of the C-terminus tail residues Y13, F14, Q17 and V19 are unchanged between SRTX-m and SRTX-m-Cter.

## Conclusions

In conclusion, despite a high homology and a very similar 3D structure, SRTX-b from *A*. *engaddensis* venom and the long-sarafotoxin, SRTX-m, from *A*. *m*. *microlepidota* venom have very different *in vivo* toxic effects. The toxic effect of SRTX-b is mainly due to a vasoconstrictor effect leading to left ventricular failure, while the effect of SRTX-m is due to right ventricular failure induced by severe bronchoconstriction. The hypothesis of *in vivo* maturation of SRTX-b is not supported by our findings. Furthermore, this study emphasizes the important role of the C-terminus extension in the *in vivo* effect of endothelin-like peptides. Further experiments are therefore needed to investigate the precise mechanism of action of SRTX-m on ET receptors *in vivo*.
